# Promising Minimally Invasive Option Emerging in the Treatment of Benign Prostatic Obstruction: Prostatic Artery Embolization

**DOI:** 10.3390/jcm14248631

**Published:** 2025-12-05

**Authors:** Rasit Dinc

**Affiliations:** INVAMED Medical Innovation Institute, New York, NY 10007, USA; rasitdinc@hotmail.com

**Keywords:** benign prostatic obstruction, prostatic artery embolization, minimally invasive therapy, lower urinary tract symptoms, TURP, HoLEP, embolic agents

## Abstract

Prostatic artery embolization (PAE) has emerged as a minimally invasive treatment for benign prostatic obstruction (BPO), offering clinically meaningful symptom improvement with a favorable perioperative safety and sexual function profile. This narrative review synthesizes the current evidence on PAE relative to transurethral resection of the prostate (TURP) and holmium laser enucleation (HoLEP), and other minimally invasive surgical treatments (MISTs). PAE generally offers a more favorable perioperative safety profile and shorter recovery time, at the cost of higher reintervention rates. PAE improves lower urinary tract symptoms, quality of life, and urinary flow; however, the magnitude of improvement is generally smaller than that observed in comparative studies with TURP and HoLEP. At the same time, PAE is consistently associated with fewer perioperative complications, shorter recovery time, and a significantly higher preservation of ejaculation function. Reintervention rates after PAE are significantly higher than those after TURP, reaching approximately one in five patients at 2 years and nearly half at 5 years in long-term randomized follow-up, suggesting limited long-term durability compared with resective surgery. This review summarizes current patient selection criteria, anatomic and technical considerations, embolization material choices, and clinical outcomes, and also presents comparative data with TURP, HoLEP, and GreenLight photoselective vaporization. Emerging technologies, including imaging guidance and AI-assisted planning, may further optimize patient selection and procedural consistency, but longer-term comparative trials and standardized protocols are needed. Overall, PAE offers an option for carefully selected patients who prioritize functional preservation or are at high surgical risk, with the added disadvantage of lower long-term durability compared to standard surgical approaches.

## 1. Introduction

Benign prostatic obstruction (BPO), usually caused by benign prostatic hyperplasia (BPH), is a highly prevalent condition in aging men, with a prevalence of up to 80% in men over 70 [1]. BPO causes a range of lower urinary tract symptoms (LUTS), including increased urinary frequency, urgency, weak stream, nocturia, and incomplete evacuation, significantly impairing quality of life and increasing healthcare burden. In the United States alone, annual direct costs associated with BPH-related complications exceed $4 billion [1,2].

Management of BPO generally follows a stepwise progression: from conservative observation and pharmacological treatment to surgical interventions such as transurethral resection of the prostate (TURP), which remains the gold standard for patients unresponsive to medical therapy [3]. However, TURP and other surgical options, such as holmium laser enucleation of the prostate (HoLEP) or open prostatectomy, are associated with significant perioperative risks, such as bleeding, retrograde ejaculation, erectile dysfunction, and prolonged hospital stays [4]. Despite their effectiveness, a significant portion of patients seek alternatives that better preserve ejaculatory function, reduce postoperative morbidity, and provide faster recovery. This unmet need has accelerated interest in minimally invasive options such as prostatic artery embolization (PAE). PAE has evolved over time from an experimental treatment to an evidence-based treatment endorsed by major guidelines, including the 2023 European Association of Urology and the 2023 American Urological Association recommendations [3,5].

Since the first clinical application in the early 2010s, PAE has gained increasing interest due to its outpatient applicability, low complication profile, and potential to preserve sexual and urinary function. However, this approach has limitations such as higher reintervention rates than traditional surgery (up to 20% within two years), limited long-term durability data, and a lack of standardization of embolic agents and patient selection criteria [6,7,8].

This study aims to provide a comprehensive overview of PAE in the management of BPO, with emphasis on anatomical considerations, procedural techniques, available embolization materials, clinical efficacy, safety profile, and its role within the broader spectrum of minimally invasive therapies.

## 2. Methodology

This article is a narrative review, not a formal systematic review. A structured, topic-focused search strategy was used to identify relevant clinical studies, technical reports, guidelines, and meta-analyses, but formal risk of bias or quality scores (e.g., ROBINS-I, AMSTAR) were not calculated.

### 2.1. Search Strategy

A targeted literature search was performed in PubMed, Embase, and Cochrane Library from database inception to the time of manuscript preparation, to June 2025. The core search terms included “prostate artery embolization”, “PAE”, “benign prostatic hyperplasia”, “benign prostatic obstruction”, “lower urinary tract symptoms”, “transurethral resection of the prostate”, “holmium laser enucleation”, “GreenLight laser”, and “minimally invasive therapy”. Searches were limited to English-language publications involving human subjects. Reference lists of key articles and contemporary guideline documents were hand-searched to identify other relevant publications.

### 2.2. Study Selection Approach

Studies were selected pragmatically to reflect the current evidence base and clinical practice. Priority was given to (1) randomized controlled trials (RCTs) comparing PAE with TURP, sham procedures, or other MISTs; (2) prospective and retrospective cohort studies with larger sample sizes (≥100 patients), and ≥12-month of follow-up; (3) systematic reviews and meta-analyses comparative results; and (4) guideline or consensus documents from major professional societies. Included studies were required to report at least one validated outcome, such as International Prostate Symptom Score (IPSS), maximum flow rate (Qmax), quality of life (QOL) scores, or retreatment rates, and to provide a sufficient technical explanation for interpreting the embolization method.

Given the diversity of study designs, findings were synthesized narratively rather than aggregated statistically.

**Figure 1:**
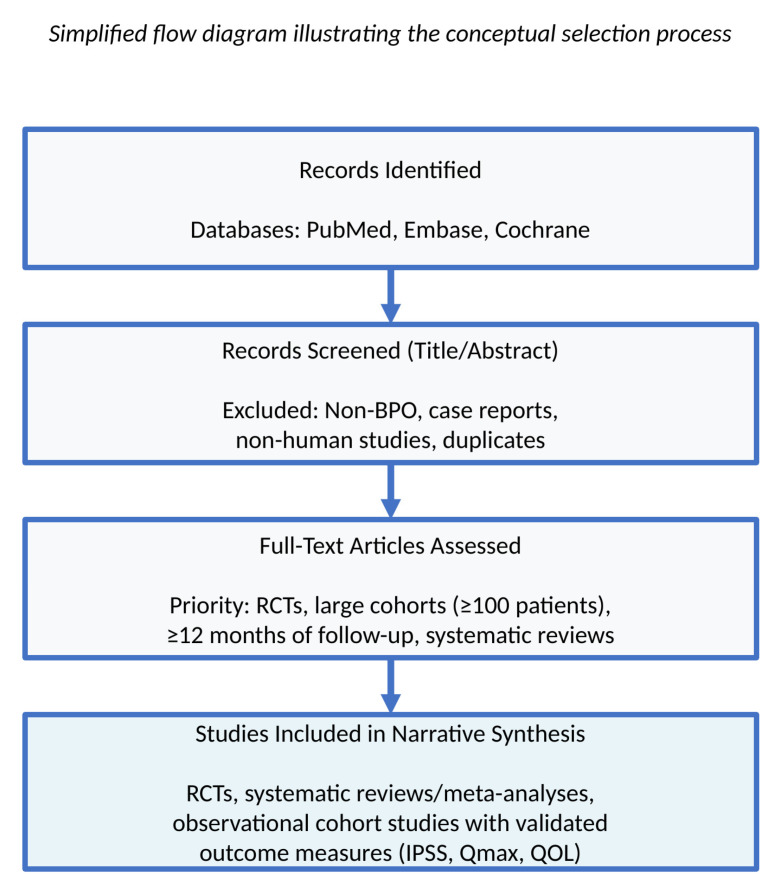
Literature Selection Process. The simplified flow diagram of the narrative review represents the conceptual selection process for a narrative review. Numerical figures are not presented because formal PRISMA methodology was not used.

A simplified flowchart is provided in the figure to illustrate the conceptual selection process (records identified → screened → assessed for eligibility → included in narrative synthesis), but exact numerical counts are not presented as this review was not conducted as a formal PRISMA-based systematic review (Figure 1).

## 3. Patient Selection and Clinical Decision Making

### 3.1. Candidate Selection Criteria

The clinical success of PAE depends on appropriate candidate selection and detailed knowledge of pelvic artery anatomy, as variations and tortuosity can make catheterization difficult [9,10]. Ideal candidates include men with moderate to severe LUTS (IPSS ≥ 15) who are resistant or intolerant to medical therapy, those seeking ejaculatory protection or outpatient treatment, those unfit for surgery, those with a prostate volume of ~30–200 mL, and those with catheter-related retention when surgery is delayed or contraindicated [11,12].

Relative contraindications include unsafe arterial access, active UTI, bladder atony or neurogenic dysfunction, very large prostates (≥200 mL), and suspected or confirmed prostate cancer. Anticoagulation can be managed with coordinated care [13]. For very large glands, PAE remains an option with counseling regarding retreatment expectations [14,15].

Compared to TURP, PAE offers fewer complications and better ejaculatory preservation, while offering smaller improvements in IPSS and Qmax and higher retreatment rates.

### 3.2. Rationale for Clinical Decision Making

PAE alleviates BPO by selectively occluding arterial blood flow to the transition zone, inducing ischemic contraction, and reducing outflow obstruction. It protects the capsule and sphincter while minimizing incontinence and ejaculatory dysfunction [16,17,18]. Preprocedural computed tomography angiography (CTA) and magnetic resonance angiography (MRA) are essential for mapping the vascular anatomy and preventing off-target embolization [7,15]. Intraprocedural cone-beam CT (CBCT) also provides real-time, high-resolution guidance to enable highly selective catheterization. Symptom burden and outcomes should be monitored with IPSS and QoL scores [19].

## 4. Technical Considerations: Clinically Relevant Aspects

### 4.1. Procedural Overview

**Figure 2:**
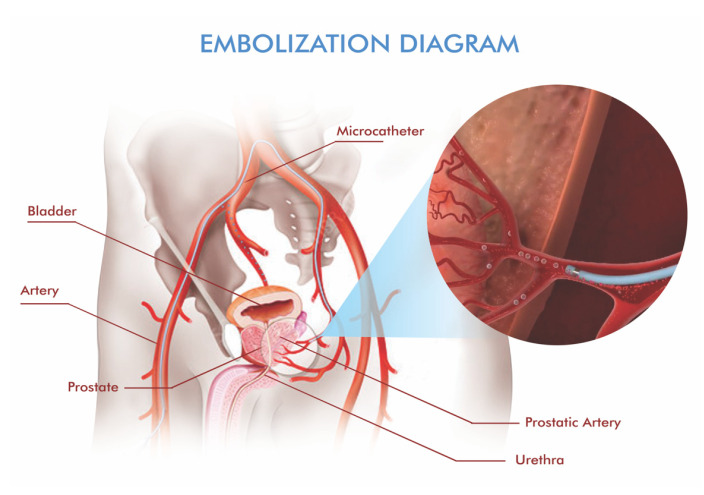
Schematic depiction of prostatic artery embolization. A microcatheter is advanced super-selectively into the prostatic arterial branches, and embolic material is delivered to reduce hyperplastic tissue while avoiding non-target territories.

PAE is typically performed as an outpatient procedure under local anesthesia with optional light sedation. Transfemoral or transradial access is usually used for vascular access. For super-selective access, a microcatheter is advanced into the prostatic arterial branches under fluoroscopic guidance [15,20]. Real-time angiography and CBCT are crucial at this stage to identify anatomic variants and prevent off-target embolization of the bladder, rectum, or penile vasculature [18] (Figure 2).

Bilateral embolization should be performed whenever technically feasible, as unilateral procedures are associated with inferior outcomes due to compensatory collateral flow, and may require staged contralateral embolization [9,15].

### 4.2. Embolic Agents and Selection Criteria

Various types of embolic agents are available for PAE, and their selection is influenced by vascular anatomy, procedural complexity, and operator preference. Based on available systematic reviews, particles of 300–500 μm are most frequently recommended as a balance between efficacy and safety [13]. Smaller particles (100–300 μm) allow deeper penetration into the transition zone and may enhance prostate devascularization, but some studies have reported a higher risk of ischemic irritation to adjacent organs and therefore recommend their use with caution. A recent prospective series of PAE using small-caliber beads (150–300 μm) demonstrated significant IPSS and Qmax improvements with low major complication rates, suggesting that small-particle protocols are feasible in experienced hands [21]. Larger particles (>500 μm) appear less effective in providing symptom relief and are rarely used as stand-alone agents [13]. Calibrated spherical microspheres (Embosphere, Bead Block) provide predictable distribution and are preferred over irregular PVA particles (20).

The dual-size polyvinyl alcohol (PVA) protocol is an alternative approach. This protocol recommends a sequential approach of 100 μm (2–5 mL) followed by 200 μm to prevent distal migration while maintaining proximal penetration [13,20]. PVA is cost-effective but exhibits greater variability in results compared to calibrated microspheres.

Liquid embolic agents such as n-butyl cyanoacrylate (NBCA) and ethylene-vinyl alcohol copolymer (EVOH) provide deep and sustained penetration [22]. NBCA and EVOH (Onyx) require expertise and are reserved for challenging anatomy or failed particle embolization. High dilution ratios (1:5 to 1:8 NBCA:Lipiodol) are critical to prevent reflux and off-target embolization [19]. These agents are being investigated for primary PAE and are reserved for specific scenarios (particularly complex or collateralized anatomy) that require greater technical expertise [20].

### 4.3. Special Populations and Technical Modifications

Technical success in contemporary series is generally high, exceeding 85% in the standard cohorts, but tends to be lower in large prostates (≥200 mL). Some authors have investigated smaller particles and staged embolization for very large glands, but robust data on long-term durability remain limited, particularly for prostates >5 years and ≥150. Current series suggest lower clinical success and higher retreatment in this subgroup compared with smaller glands, and therefore many centers still prefer primary surgery for very large prostates [13,23].

Outcomes are favorable in patients with urinary retention, with catheter removal achieved within 2–4 weeks after PAE in 82–94% of patients. Bilateral embolization, prostate volume >50 mL, and intact bladder function are predictors for successful voiding recovery [24].

Comorbidities should also be considered. Anticoagulants are not a contraindication but require coordinated periprocedural management. Evidence in patients with renal failure or cirrhosis remains limited, and young men (<55 years) are underrepresented, making long-term durability uncertain [20].

## 5. Clinical Outcomes and Comparative Effectiveness

Where appropriate, we indicate the predominant type of evidence (randomized controlled trial, observational cohort, or meta-analysis) for each key outcome and comparative tables. A growing number of comparative studies have examined the efficacy and safety of PAE compared to TURP, which has long been the gold standard for treating BPO. While both interventions aim to improve LUTS, their clinical profiles differ in terms of symptom relief, functional preservation, complication rates, and long-term durability.

### 5.1. PAE vs. TURP: Critical Analysis

Randomized evidence suggests that TURP provides greater mean improvements in symptoms and urinary flow compared to PAE at 1–2 years [25,26,27,28]. In a two-year randomized controlled trial, TURP led to a significantly greater reduction in IPSS compared to PAE (12.09 vs. 9.21 points, *p* = 0.047), as well as more significant improvements in Qmax (10.23 vs. 3.9 mL/s, *p* < 0.001) and post-void residual volume (204 vs. 62 mL, *p* = 0.005) [6]. Five-year follow-up of this study showed that PAE maintained clinically meaningful symptom relief, but the magnitude of improvement in IPSS and Qmax remained smaller than after TURP [25]. These findings are consistent with a systematic review and meta-analysis by Knight et al. [7], which pooled data from 1044 patients and showed that while TURP provided slightly greater improvements in IPSS and Qmax, PAE was associated with fewer adverse events, particularly related to sexual function and length of hospital stay. These findings are consistent with a more recent updated systematic review that synthesized contemporary PAE series and confirmed that PAE provides clinically meaningful symptom relief with a favorable safety and sexual function profile, and acknowledged higher retreatment rates compared to TURP and other resective procedures [29]. A summary of the major clinical studies evaluating PAE, including embolizing agents, symptom improvement, and complication rates, is presented in Table 1.

Randomized studies, including a sham-controlled design, have confirmed the true therapeutic benefit of PAE [16,17,33]. Comparative evidence is limited by several methodological issues. Most randomized trials include highly selected patients treated in specialized centers, limiting generalizability. Furthermore, as reflected in practice documentation, there is significant technical heterogeneity, ranging from pre- and intraprocedural imaging strategies to microcatheter position, particle size, and even embolism class [15,20]. Outcomes are also inconsistently defined and timed; studies differ in how they report IPSS, Qmax, PVR, and ejaculatory endpoints, preventing robust pooling.

### 5.2. Sexual Function Preservation: PAE’s Primary Advantage

**Figure 3:**
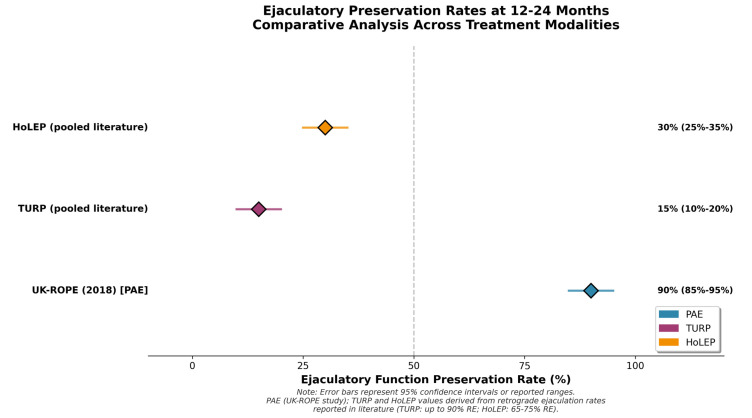
Ejaculatory preservation rates at 12-24 months in representative studies of PAE, TURP, HoLEP, and GreenLight PVP. Error bars represent 95% confidence intervals where reported. Circles represent individual study estimates; diamonds represent compiled or meta-analytic summaries from published sources. PAE consistently demonstrates greater preservation of ejaculatory function compared to resective procedures.

PAE preserves ejaculatory function more often than resective surgery. A 2022 meta-analysis by Wang et al. found lower retrograde ejaculation rates and better preservation of erectile function compared to TURP in PAE patients [26]. Retrograde ejaculation is reported in up to 90% of patients after TURP [28]. The UK-ROPE study (2018) and other prospective studies report that ejaculation preservation is achieved in 85–95% of patients undergoing PAE [34]. Ghahfarokhi et al. reported positive ejaculation preservation in a conference abstract representing preliminary non-peer-reviewed data (mean difference 1.81; 95% CI: 1.25–2.37) [28]. This favorable sexual profile represents the most consistent clinical advantage of PAE. Figure 3 shows a visual comparison of ejaculatory preservation in representative studies, demonstrating the consistent superiority of PAE over TURP and HoLEP.

### 5.3. Safety and Complication Profile

PAE is associated with a lower perioperative morbidity burden and mostly minor side effects (e.g., transient dysuria, hematuria, pelvic discomfort). The minor complication rate is seen in around 5–10% of patients and generally resolves spontaneously [30,31]. Serious off-target embolization is rare in experienced centers [20]. In contrast, TURP carries risks of bleeding, transfusion, and retrograde ejaculation, as well as rare but known complications such as TUR syndrome [18]. Therefore, the clear safety profile favors PAE in the immediate postoperative period, particularly regarding sexual function, while TURP provides greater functional gain.

### 5.4. Reintervention Rates: A Critical Limitation

Reintervention rates after PAE remain a significant limitation. Up to 20% of patients undergoing PAE required TURP within two years due to unsatisfactory clinical results [6]. Reintervention rates vary according to prostate size, completeness/bilaterality of embolization, and center experience. Factors associated with a higher risk of retreatment include large baseline volume, unilateral or incomplete embolization, and complex vascular anatomy [15]. Table 2 presents a structured comparison of key outcome measures between PAE and TURP, synthesizing data from major studies.

Overall, RCT data demonstrate superior efficacy of TURP compared to PAE: IPSS reduction of −18 to −20% compared to −12 to −15% for PAE; Qmax improvement of +15–18 mL/s compared to +6–8 mL/s. Advantages of PAE include lower major complications (1–3% compared to 10–15%), better sexual protection (5–10% compared to 65–80% for retrograde ejaculation), and faster recovery [6,25,27] 2-year reintervention rates favor TURP (3–5% compared to 10–15% for PAE), but this difference narrows over time [6,24] Five-year results from a Swiss RCT confirm these trends, with cumulative PAE reintervention rates reaching 47% [6].

Studies directly comparing PAE with other MISTs are limited. Section 5.5 provides some examples that comparing PAE and other MISTs. Table 3 shows the 12-month comparative results of PAE and other minimally invasive approach treatments in BPO.

### 5.5. Comparison with Other Minimally Invasive Treatments (MISTs)

**PAE vs. HoLEP**: Recent randomized and comparative studies, including the HoPE trial, demonstrate that both PAE and HoLEP provide similar short-term symptom relief, with similar 3-month IPSS reductions (−20 vs. −17, *p* = 0.24) and improvements in quality of life (−3 to −4). However, HoLEP provides greater objective improvements in urinary flow (Qmax +12.8 vs. +6.7 mL/s, *p* = 0.02) and prostate volume reduction, reflected in greater PSA reductions (70% vs. 35%) due to tissue resection rather than ischemic shrinkage. Erectile function outcomes consistently favor PAE. In the HoPE study, mean IIEF-15 improvement was +5 for PAE versus −7 for HoLEP (*p* = 0.047), and continence outcomes also favored PAE (*p* = 0.013), with anterograde ejaculation almost universally preserved [27,38]. At 12 months, both techniques maintained similar symptom and quality of life improvements (IPSS-17 versus −15), but overall complication rates remained higher after HoLEP (28% versus 10%; RR 3.19, *p* = 0.009) [38]. For large prostates (>80 mL), long-term outcomes show better durability for HoLEP or the combined PAE + HoLEP approach (5-year success: 86% versus 48% for PAE alone), while prostates ≥150 mL generally prefer primary surgery [6,12]. In addition, a recent propensity score-matched study comparing en bloc HoLEP with robotic-assisted simple prostatectomy in large prostates confirmed that HoLEP offers excellent perioperative safety and short-term functional outcomes, further supporting its role as a permanent reference standard in surgically suitable patients [40].

**PAE vs. GreenLight Laser Therapy**: Direct head-to-head comparisons are limited. Network meta-analyses show similar symptom improvement over 1–2 years [25,30]. GreenLight photoselective vaporization (PVP) provides more rapid symptom relief (months rather than weeks) but is associated with more transient dysuria, whereas PAE provides milder improvement and excellent preservation of sexual function. Both outperform TURP and HoLEP in preserving ejaculation, but confirmatory RCTs are needed [18] (Box 1).

Box 1 **Clinical Decision Framework**

- TURP: Maximizes efficacy and durability; has acceptable sexual side effects; is cost-effective.
- HoLEP: Suitable for all prostate sizes; has durable outcomes; has higher sexual side effects and operator dependency.
- PAE: Ideal for patients prioritizing sexual preservation, outpatient management, or unsuitable for surgery; accepts a higher risk of reintervention.
- GreenLight PVP: Balances efficacy and recovery; suitable for moderate-sized prostates and patients taking anticoagulants.

## 6. Critical Limitations and Biases

Despite increasing clinical evidence, several methodological and practical limitations continue to influence the interpretation of PAE results. Significant technical heterogeneity exists across studies, including differences in embolic particle size (100–700 μm), embolization endpoints (near-stasis and constant volume), and imaging protocols (DSA and CBCT). These differences contribute to inconsistent efficacy and safety results and hinder data pooling. Adopting standardized procedural protocols will improve reproducibility and enable more robust meta-analyses [20].

Another limitation is long-term loss to follow-up: 30–50% loss after three years has been reported. Such loss rates may introduce survival bias by primarily excluding treatment failures and may overestimate clinical durability [24,36]. Intention-to-treat analyses or estimation methods for missing data are rare in this literature.

Publication and selection biases also persist because most data are derived from specialized, high-volume centers with early experience. Funnel plot asymmetry in meta-analyses suggests small study effects favoring favorable outcomes [25]. Larger registry-based or population-level data are needed to reflect real-world practice.

The operator dependence of the procedure further limits generalizability. Achieving consistent bilateral embolization success typically requires 30–50 cases, and results from specialized centers may not be transferable to community settings [20].

Potential conflicts of interest are another concern, as many published studies involve interventional radiologists with consulting or research relationships with device manufacturers. While generally disclosed, as in this article, such specialized affiliations should be considered when comparing PAE with procedures performed by urologists.

Finally, important evidence gaps remain. Data are limited for patients with very large prostates (≥200 mL), those with significant comorbidities such as end-stage renal disease or cirrhosis, and younger men (<55 years) for whom long-term durability is uncertain. Cost-effectiveness analyses are still preliminary and are highly dependent on institutional and regional factors [13,20].

Operator experience also influences reported outcomes. Early PAE series, particularly those published before the widespread adoption of cone-beam CT and advanced navigation techniques, reported lower technical success and higher retreatment rates. Contemporary cohorts with more procedural experience demonstrate improved bilateral embolization rates and more consistent clinical outcomes. Bilateral completion is particularly important because incomplete or unilateral embolization has been associated with higher rates of clinical failure and subsequent reintervention. These factors should be considered when interpreting differences between studies.

## 7. Future Directions

Advances such as improved microcatheters, real-time cone-beam CT, and shape-memory polymer occlusive devices can improve consistency and safety; rigorous evaluation against patient-centered outcomes is required [41]. Artificial intelligence can potentially reduce intercenter variability by supporting patient selection, anatomic mapping, risk stratification, and outcome prediction [42].

Standardized urology-interventional radiology pathways for selection, consent (open PAE-TURP trade-offs), and predefined salvage strategies (repeat PAE or surgery) should be integrated to improve outcomes and patient experience.

In select scenarios, sequential or combination approaches, such as PAE followed by surgery for persistent obstruction, may offer personalized solutions aligned with anatomy and patient priorities; prospective evaluation is necessary.

Research priorities include: (1) Long-term RCTs (5–10 years) comparing PAE with HoLEP and other MISTs; (2) technical guidelines achieved through consensus through professional societies; (3) predictive biomarkers for durability; (4) cost-effectiveness modeling across health systems; (5) real-world registry data capturing community practice outcomes; and (6) quality of life instruments sensitive to PAE-specific recovery profiles [2,13].

## 8. Conclusions

Prostatic artery embolization has become an important minimally invasive alternative for selected men with benign prostatic obstruction. Its most consistent advantages include a low perioperative morbidity profile and excellent preservation of sexual and ejaculatory function compared with resective procedures. At the same time, randomized and comparative studies have shown that symptom and flow improvements are generally less than those after TURP or HoLEP, and long-term durability remains a major limitation of PAE. Reintervention rates of up to 20% at 2 years and cumulative failure rates approaching 50% at 5 years in long-term randomized follow-up highlight the need for clear patient counseling regarding the possibility of retreatment.

Within a shared decision-making framework, PAE should be considered for patients who prioritize ejaculatory preservation, outpatient management, and perioperative risk reduction, or who are not suitable candidates for resective surgery, provided they endure higher reintervention rates. Optimized embolization materials, standardized imaging and technical protocols, and ongoing research on predictors of durability and cost-effectiveness across healthcare systems will be important to further define the role of PAE in contemporary BPO management.

## Figures and Tables

**Table 1:** Summary of key clinical studies on prostatic artery embolization (PAE).

Study Reference	Design	Sample Size	Embolic Agent	Comparator	Baseline PV (mL)	IPSS Improvement	QoL Improvement	Complication Rate	Follow-Up (Months)
[30]	Multicenter Cohort	1015	Microspheres, PVA	None	96 ± 24.7	↓ from 22 to 10	Significant (↓ from 4.3 to 2.2)	5% minor, <1% major	24
[16]	RCT	103	Microspheres	TURP	51.2 ± 16.5 (US)/52.8 ± 32.0 (MRI)	↓ 9.2 points vs. ↓ 12.1 points TURP	Comparable	PAE lower	24
[17]	RCT	60	Microspheres	TURP		Comparable to TURP	65% improvement	4% minor	12
[19]	Prospective	50	NBCA	None	98.3 ± 40.2	55.7% improvement from baseline	55.1% improvement	2% minor	36
[31]	Observational	30	EVOH	None	84 ± 40	61% improvement from baseline	64% improvement	10% minor	6
[32]	Meta-analysis	1832 (pooled)	Various (Microspheres, PVA)	TURP, others	NA	No significant difference	Comparable	Fewer adverse events with PAE	12–24
[25]	Prospective, multicenter cohort	1075	Various (Microspheres, PVA, EVOH)	None	NR	Consistent and significant (↓ from 22.6 to 10.4)	Consistent and significant	0.65% serious AEs	60
[7]	Meta-analysis	1044 (pooled)	Various	TURP	NA	(↓ IPSS (slightly less than TURP)	Comparable	Fewer adverse events with PAE	12–24
[33]	RCT	80	Microspheres	Sham	63.5 [55.5–100.0] (TRUS)/68.5 [58.0–103.5] (MRI)	↓ from 8.3 to 1.4	Significant	5% minor	12

Abbreviations: RCT, randomized controlled trial; IPSS, International Prostate Symptom Score; QoL, quality of life; PVA, polyvinyl alcohol; NBCA, N-butyl cyanoacrylate; EVOH, ethylene-vinyl alcohol copolymer; TURP, transurethral resection of the prostate; PV, prostate volume; US, ultrasound; MRI, magnetic resonance imaging; ↑, increase; ↓, decrease.

**Table 2:** Comparative results of prostatic artery embolization (PAE) and transurethral resection of the prostate (TURP).

Outcome Measure	PAE	TURP	Statistical Significance	Data Source Type	References
Baseline PV (mL)	51.2 ± 16.5 (US)/52.8 ± 32.0 (MRI)	52.1 ± 18.6 (US)/56.5 ± 31.1 (MRI)	-	RCT	[6,16]
Improvement in symptom score (IPSS)	Moderate (IPSS ↓ 9.2 at ~2 years; less than TURP)	Greater (IPSS ↓ 12.1 at ~2 years)	*p* = 0.047 (in favor of TURP)	RCT	[26,31]
Qmax	Mild (↑ ~3.9 mL/s)	Significant (↑ ~10.2 mL/s)	*p* < 0.001 (in favor of TURP)	RCT	[31]
PVR	↓ ~62 mL	↓ ~204 mL	*p* = 0.005 (in favor of TURP)	RCT	[6,27]
Decrease in prostate volume	↓ ~10.7 mL	↓ ~30.2 mL	*p* < 0.001 (in favor of TURP)	Meta-analysis + RCT	[25,27]
Preservation of ejaculatory function	High preservation (~80–90%)	Low (~20–40%, high retrograde rate)	*p* < 0.001 (in favor of PAE)	Meta-analysis + Observational	[25,27]
Erectile function (IIEF-5)	Stable or slightly improved	Slight decrease in some patients	*p* = 0.032 (in favor of PAE)	Meta-analysis + Observational	[25,27]
Hospital stay duration	Shorter (<24 h in most cases)	Longer (typically 2–3 days)	*p* < 0.001 (in favor of PAE)	RCT + Observational	[6,27]
Reintervention rate (within 2 years)	Higher (~20%)	Lower (~5–10%)	*p* = 0.024 (in favor of TURP)	RCT	[6,27]
Overall complication rate	Lower (less severe adverse events)	Higher (bleeding, incontinence, stricture)	*p* = 0.008 (in favor of PAE)	Observational	[26,35]
Return to Normal Activity	1–3 days	2–4 weeks	*p* < 0.001 (in favor of PAE)	Multiple study types	Multiple sources

Abbreviations: IPSS, International Prostate Symptom Score; Qmax, maximum flow rate; PVR, postvoid residual; IIEF-5, International Index of Erectile Function-5; PAE, prostatic artery embolization; TURP, transurethral resection of the prostate; PV, prostate volume; US, ultrasound; MRI, magnetic resonance imaging; ↑, increase; ↓, decrease.

**Table 3:** Comparative outcomes of minimally invasive treatments for benign prostatic obstruction at 12 months *. Evidence types: Values are drawn from randomized controlled trials, high-quality observational cohorts, and network meta-analyses as stated in the references.

Treatment	IPSS Reduction	Qmax Δ (mL/s)	Retrograde Ejaculation (%)	Reintervention in 2 Years (%)
PAE	−12 to −15	+6 to +8	5–10	10–15
HoLEP	−15 to −17	+12 to +15	65–75	3–5
TURP	−18 to −20	+15 to +18	65–80	3–5
GreenLight PVP	−14 to −16	+10 to +13	10–20	8–12

* Values represent approximate ranges based on representative randomized trials, observational cohorts, and network meta-analyses (e.g., HoPE trial, comparative meta-analyses) [6,23,24,25,26,30,36,37,38,39]. Exact values vary between individual studies. Δ, change.

## Data Availability

The author confirms that the data supporting the findings of this study are available in the article.
